# Protocols for Extraction of miRNA from Extracellular Vesicles of Lyophilized Human Saliva Samples

**DOI:** 10.3390/ijms26072891

**Published:** 2025-03-22

**Authors:** Valquiria Quinelato, Carlos Fernando Mourão, Thalita Alves Barreto Santos, Patrícia Cataldo de Felipe Cordeiro, Leticia Ladeira Bonato, Miria Gomes Pereira, Jose Albuquerque Calasans-Maia, Jose Mauro Granjeiro, Tomoyuki Kawase, Priscila Ladeira Casado

**Affiliations:** 1 Post-Graduation Program in Dentistry, Universidade Federal Fluminense, Niteroi 24020-140, Rio de Janeiro, Brazil; valquiriaquinelato@yahoo.com.br (V.Q.); thalitaalvesodonto@gmail.com (T.A.B.S.); patricia.cataldo@hotmail.com (P.C.d.F.C.); atm.leticia@gmail.com (L.L.B.); josecalasans@id.uff.br (J.A.C.-M.); jmgranjeiro@gmail.com (J.M.G.); priscilalcasado@gmail.com (P.L.C.); 2 National Institute of Traumatology and Orthopedics, Rio de Janeiro 20940-070, Rio de Janeiro, Brazil; 3 Department of Basic and Clinical Translational Sciences, School of Dental Medicine, Tufts University, Boston, MA 02111, USA; 4 Laboratory of Cellular Ultrastructure Hertha Meyer, Biophysics Institute Carlos Chagas Filho, Universidade Federal do Rio de Janeiro, Rio de Janeiro 1941-902, Rio de Janeiro, Brazil; miria@biof.ufrj.br; 5 Division of Oral Bioengineering, Institute of Medicine and Dentistry, Niigata University, Niigata 950-8680, Japan

**Keywords:** microRNA, extracellular vesicles, exosomes, lyophilization

## Abstract

Extracellular vesicles (EVs) are emerging as crucial biomarkers in molecular diagnostics, providing early detection of disease progression. Although ultracentrifugation remains the gold standard for vesicle isolation from biofluids, it has limitations in scalability and accessibility. This study presents lyophilization as an innovative method for preserving EVs and isolating microRNAs from saliva, utilizing its proven ability to maintain biological activity and prevent unwanted chemical reactions. We assessed five different sample preparation protocols combined with a dual-purification strategy. Structural and molecular integrity analyses revealed that lyophilized samples retained essential EV characteristics, including CD63/CD9 membrane localization. QELS analysis and electron microscopy confirmed distinct vesicle populations in both ultracentrifuged (30–50 nm and 320–360 nm) and lyophilized samples (50–70 nm and 360–380 nm). Importantly, lyophilized samples exhibited higher total RNA concentrations (*p* < 0.0001) while preserving key microRNA signatures (miR-16, miR-21, miR-33a, and miR-146b) with high fidelity. The efficacy of lyophilization is linked to its ability to systematically reduce solvent content through sublimation while maintaining vesicle integrity and molecular cargo. This method offers a practical, scalable alternative for EV isolation with significant implications for biomarker-based diagnostics.

## 1. Introduction

In the intricate landscape of cellular communication, extracellular vesicles (EVs) have emerged as intriguing nano-scale messengers, orchestrating complex biological processes across nearly all mammalian cells. These remarkable structures, which range from exceedingly small particles to larger vesicles under 1000 nm, serve as advanced delivery vehicles for a diverse array of molecular cargo, including nucleic acids (mRNA, miRNAs, and other RNA species), lipids, and proteins. The EV family consists of two main populations: microvesicles (MVs)/microparticles (MPs), ranging from 100 to 1000 nm, and the smaller exosomes, measuring less than 120 nm [1,2,3].

At the core of EV-mediated communication lies a particularly intriguing component: microRNAs (miRNAs). These small but powerful non-coding RNAs, measuring just 18–25 nucleotides, primarily reside within exosomes—specialized endosome-derived vessels that facilitate both local and long-distance cellular communication. Through their remarkable ability to transport proteins, lipids, mRNA, miRNA, and DNA between cells, exosomes serve as cellular couriers. The contained exosomal RNAs significantly influence biological processes by expertly regulating gene expression through two distinct mechanisms: direct mRNA degradation and interruption of the translation process [4,5,6,7,8].

The ubiquitous nature of circulating miRNAs, whether they are contained in exosomes or in the vesicle-free form associated with carrier proteins [9,10,11,12], is particularly striking, as they can be detected across an impressive spectrum of biological fluids—from saliva and blood to cerebrospinal fluid, tears, and milk [13,14,15,16,17,18,19]. Their presence extends beyond fluids to tissues [20,21] and specific subcellular compartments, including cytoplasm, nucleus, and membrane-bound organelles [20,22,23]. This widespread distribution, combined with their stability and specificity, has positioned miRNAs as exceptional candidates for disease biomarkers, offering promising applications in diagnosing and monitoring conditions ranging from malignant neoplasms [24] and fibromyalgia [19,25] to cardiac [8,26] and autoimmune diseases [5,20,27].

In the quest to harness these powerful molecular markers, lyophilization—or freeze-drying—presents an innovative approach. This sophisticated stabilization process systematically reduces solvent content through sublimation and desorption, effectively preserving biological activity while preventing unwanted chemical reactions [28,29,30]. Since its inception in 1935, to preserve biological products [31], lyophilization has revolutionized multiple scientific fields. In pharmaceutical applications, it has proven invaluable for stabilizing therapeutic proteins [32,33], concentrating plant extracts [34], and producing diagnostic tests [35]. Notably, recent research by Neupane et al. (2021) [36] demonstrated lyophilization’s remarkable ability to maintain the crucial characteristics of bioinspired cell-derived nanovesicles during long-term storage.

Lyophilization’s versatility extends far beyond pharmaceuticals. It has essential applications in food preservation [37], agricultural biological control [38], and biocatalysis [39]. Studies particularly relevant to molecular biology have shown that lyophilized tissue samples maintained at room temperature for extended periods (14 days to 20 months) yield genomic DNA and mRNA comparable to frozen samples [40,41,42].

While various methods exist for isolating EVs—including precipitating reagents, filter columns, immunoaffinity, and density gradient techniques [7,43,44,45]—each presents unique considerations regarding cost, equipment requirements, processing time, sample specifications, and purity levels. The selection of an appropriate protocol depends heavily on sample characteristics and analytical requirements [44]. Given this context, our study seeks to evaluate the effectiveness of lyophilization as a method for preserving salivary EVs and their miRNA cargo, potentially offering a more practical approach to sample preparation prior to molecular analysis.

## 2. Results

### 2.1. Patients Information

A total of forty-four participants were included in this study. There were 28 (64%) women and 16 (36%) men, with a mean age of 43.3 ± 10.9 years. Ethnicity was determined by self-declaration: 18 (40.9%) identified as White, 15 (34.1%) as Brown, and 11 (25%) as Black. Twenty-eight (64%) reported using medications (contraceptive, antihypertensive, anti-hypothyroidism, antihistamines, and antidepressants), and 39 (90%) indicated having systemic diseases (hypertension, asthma/rhinitis/allergy, hypothyroidism, arthrosis/bursitis, psychological disorders, osteoporosis, anemia).

### 2.2. Extracellular Vesicles Characterization

**Figure 1:**
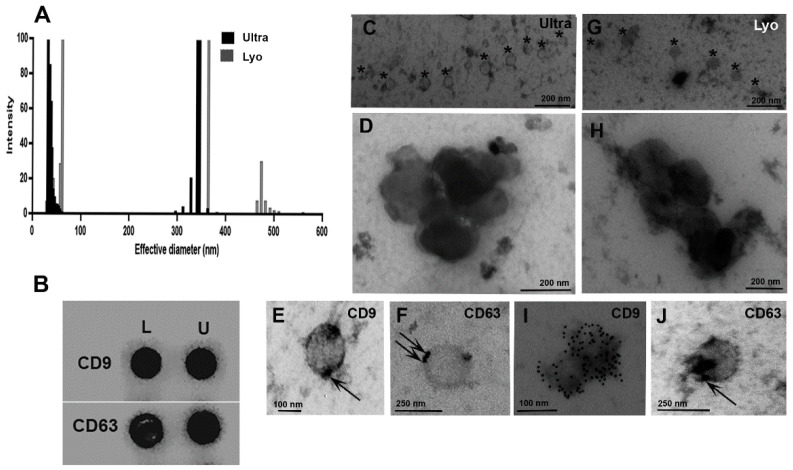
Analysis of vesicles from saliva under different isolation conditions. (**A**) QELS analysis of vesicles in suspension comparing ultracentrifugation (black column) and lyophilization (gray column) methods, with particle distribution analyzed at higher intensities. Vesicles from the ultracentrifugation method are divided into two size populations (30–50 nm and 320–360 nm), while those from lyophilization are categorized into three populations (50–70 nm, 360–380 nm, and 470–510 nm). (**B**) Dot blotting analysis using 25 µg of protein per well. Immunolocalization of specific EV proteins was evaluated using the indicated antibodies. (**C**–**F**) EVs from ultracentrifugation and (**G**–**J**) from lyophilization procedures. Micrographs display several EVs of varying sizes, sometimes appearing to adhere to one another. (**C**,**G**) The subpopulation of small EVs is marked by an asterisk line. (**D**,**H**) Larger EVs from Ultra or Lyo fractions, respectively. (**E**,**F**,**I**,**J**) The arrows indicate the immunolocalization of CD9 or CD63 markers in isolated EVs via electron microscopy. Notable CD9 localization is observed in the Lyo fraction in the image (**I**)—legend: Ultra—ultracentrifugation; Lyo—lyophilization.

QELS analysis of EVs in suspension, comparing the two methods, reveals the particle distribution analyzed at higher intensities (Figure 1A). The positive dot blotting analysis was conducted using 25 µg of protein per well (Figure 1B). Electron microscopy analysis of EVs isolated from supernatant saliva during lyophilization (Figure 1C–F) and ultracentrifugation (Figure 1G–J) shows similarities in size and particle distribution. The arrows indicate CD63 and CD9 localization on the EV membrane in both groups (Figure 1E,F,I,J).

### 2.3. Comparison of Protocols

**Figure 2:**
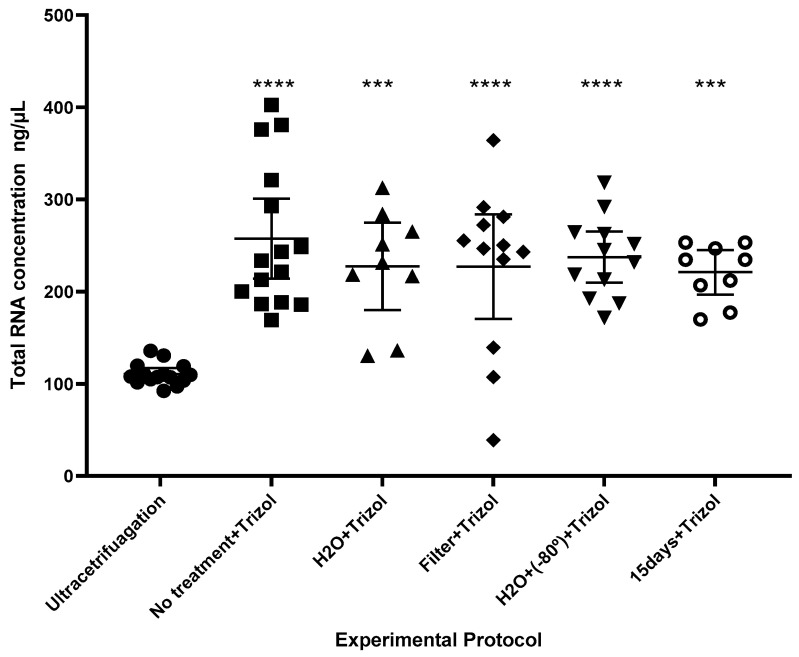
Effect of experimental protocol on the amount of total RNA (ng/µL) recovered. Data are expressed as mean and 95% confidence interval (vertical bars). The statistical significance related to ultracentrifugation protocol: *p* < 0.001 (***) and *p* < 0.0001 (****). (Samples were collected from participants 1, 2, and 3) (ANOVA with Tukey post hoc).

The comparison between different sample preparation protocols, the lyophilized saliva supernatant, followed by extraction of the total RNA by the Trizol method and submission to the second purification method, showed statistical differences between the groups, *p*-value < 0.0001 (Figure 2).

**Figure 3:**
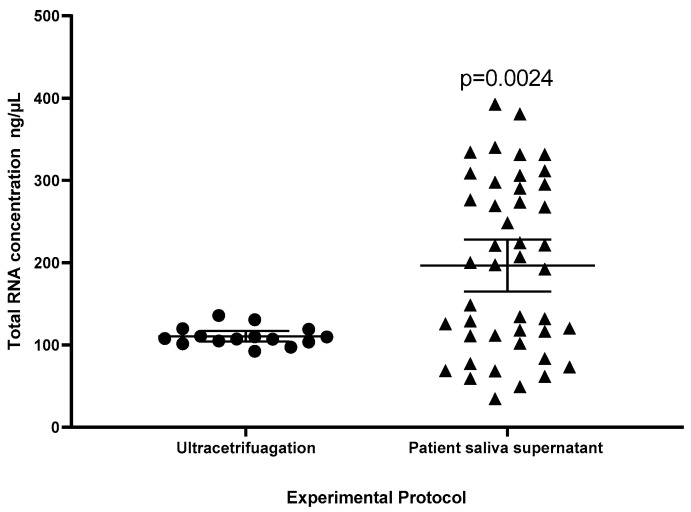
Effect of experimental protocol on the amount of total RNA (ng/µL) recovered. Data are expressed as mean and 95% confidence interval (vertical bars). Statistical significance was observed in the comparison of both groups (unpaired *t*-test).

A total of 44 patient samples were collected, lyophilized, and isolated by the Trizol method to assess the lyophilization process on many samples and analyze the amount of total RNA (ng/µL) recovered compared to the ultracentrifugation group. A statistical difference was observed between the ultracentrifugation group and the Lyophilized Patient Supernatant (*p* = 0.0024) (Figure 3).

The purity analysis of the total RNA was performed by spectrophotometry nanodrop. The results demonstrated the high purity of the isolated samples. All experimental groups showed a 260/280 correlation higher than 1.80 (±0.15) and a 260/230 correlation higher than 2.13 (±0.10) (Table 1).

No treatment + Trizol (five independent experiments); filtered saliva supernatant (0.2 µm filter) and lyophilization = Filter + Trizol (four independent experiments); H_2_O + (−80 °C) + Trizol (four independent experiments); H_2_O + Trizol and 15 days + Trizol (three independent experiments). Data are presented by means followed by the standard deviation (SD).

### 2.4. Bioinformatic Analysis

The bioinformatic analysis (URL: http://mirwalk.umm.uni-heidelberg.de accessed on 15 October 2024) showed a close relationship between the genes involved in bone homeostasis with the miRNAs (miR-21, miR-33a, and miR-146b) which were selected for this study. The miR-21 presented a relationship with the gene Receptor activator of nuclear factor κ B *(RANK*), also known as tumor necrosis factor ligand superfamily member 11A (*TNFRSF11A*). The miR-146b presented a relationship with the *TRAF6*, *RUNX2*, and *STAT3* genes. The miR-33a presented several binding sites with the gene *Special AT-rich sequence-binding protein 2* (*SATB2*) (Table 2).

### 2.5. Analysis of microRNA in Lyophilized and Ultracentrifuged Saliva Supernatant Through Real-Time PCR

**Figure 4:**
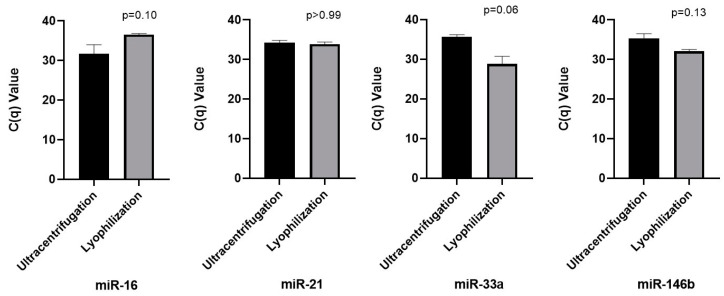
Real-time PCR analysis of the microRNA isolated from lyophilized and ultracentrifuged saliva supernatant optimized with phenol/chloroform: miR-16 (endogenous control), miR-21, miR-33a, and miR-146b. N = 2 (participants 1 and 2), TaqMan Assay was run in duplicate. Data are expressed as median and 95% confidence interval (horizontal bars). There is no statistical significance in comparing the two EVs isolation methods for each microRNA (Mann–Whitney test). The (no treatment + Trizol) group samples were used for the lyophilization.

The presence of miR-16 (endogenous control), miR-21, miR-33a, and miR-146b in the total RNA isolated from lyophilized and ultracentrifuged saliva supernatant was confirmed by Real-Time quantitative PCR. No statistical differences were shown between lyophilized and ultracentrifuged samples for assessed microRNA (Figure 4).

## 3. Discussion

The lyophilization process reduces the substance solvent without impairing its structural, biological, and functional features [33,36]. This method also prepares pharmaceutical formulations containing active, structurally complex components and drug delivery systems [32,33,34,35]. Furthermore, industrial foods use freeze-drying to preserve the food’s nutritional properties [37]. This study aimed to assess if it is possible to extract microRNA (miRNAs) from lyophilized biofluids. The results showed that CD63/CD9 localization on the EV membrane was analyzed through electron microscopy analysis. Furthermore, QELS analysis and electron microscopy revealed distinct populations of vesicles in the ultracentrifuged samples (30–50 nm and 320–360 nm) and the lyophilized samples (50–70 nm and 360–380 nm). This characterization of subpopulations of extracellular vesicles (EVs) is consistent with descriptions found in the literature [1,2,3]. The qPCR analysis confirmed the amplification of miRNAs (miR-16, miR-21, miR-33a, and miR-146b), demonstrating lyophilization as an innovative approach for preserving EVs and isolating microRNAs from saliva supernatant.

Additionally, the apparent discrepancy between QELS analysis and EM data in vesicle size distributions reflects methodological limitations rather than true biological differences. While QELS showed distinct size populations, its intensity-based measurements can artificially emphasize size differences. The EM data (Figure 1C–J) reveals a continuous size distribution for both isolation methods, with vesicles spanning from ~30 nm to >400 nm. This continuous distribution better represents the biological reality of EV populations. Importantly, both methods preserved EV integrity, as evidenced by CD63 and CD9 immunolabeling (Figure 1E,F,I,J). The additional larger population seen in lyophilized samples (470–510 nm) likely represents aggregates formed during the freeze-drying process, a known limitation of this technique that does not significantly impact vesicle functionality [46].

The exosomal location of miRNAs requires processes for isolating extracellular vesicles before miRNA extraction. The gold-standard method for vesicle isolation in biofluids is ultracentrifugation [44,47,48]. However, new methods have now been introduced, such as precipitation reagents [7,43,49], filter columns, size exclusion columns [44], and lipid-nanoprobe systems [50]. These techniques present additional costs; none have been appropriately described for salivary samples, and some are without indication for nucleic acid analysis. The great advantage of lyophilization is its low cost and simplicity.

While ultracentrifugation isolates EV-contained miRNAs, lyophilization likely captures both EV-associated and free miRNAs. The literature suggests that salivary miRNAs are preferentially encapsulated within EVs [10,12], with free miRNAs representing a minor fraction [10]. Although we did not quantify free miRNAs in the EV-depleted supernatant, their contribution is likely negligible, supporting lyophilization as a robust method for total miRNA analysis in saliva.

This work showed a higher yield of total RNA isolated from lyophilized saliva supernatant (233.50 (±78.26) ng/µL) compared to ultracentrifuged samples (110.50 (±11.65) ng/µL), *p* < 0.0001. The higher total RNA yield in lyophilized samples (Figure 3) likely results from capturing both EV-contained and free RNA, unlike ultracentrifugation, which isolates only EV-associated RNA. This is consistent with reports that salivary miRNAs are predominantly EV-encapsulated, with a minor free fraction [10] suggesting lyophilization enhances total RNA recovery.

Another advantage of lyophilization is the possibility of long-term storage of lyophilized samples at room temperature and subsequent RNA/DNA isolation [37,38,39]. This work showed no difference between isolated RNA yield performed immediately after lyophilization (no treatment + Trizol group) and storage for 15 days at room temperature (15 days + Trizol groups), *p*-value > 0.99. Additionally, some sample preparation protocols were implemented before or after the saliva supernatant lyophilization process, and there was no statistical difference in the yield of the samples in the groups studied (no treatment + Trizol) compared to (H_2_O + Trizol, Filter + Trizol, and H_2_O + (−80 °C) +Trizol), *p*-values > 0.99.

In addition to the amplification for some microRNAs and in contrast with the results of the study by Lam et al. (2012) [51], which states that salivary samples do not reach a rigid standard of quality (260/280 ratio between 1.8 and 2.2), the lyophilized saliva supernatant samples in the present study exhibited 260/280 ratios above 1.82 (±0.04), indicating their high quality. On the other hand, the ultracentrifuged saliva supernatant showed values of 1.73 (±0.01). However, Zocco and Zarovni (2017) [47] considered a 260/280 ratio above 1.60 to be optimal for biofluids. Therefore, after applying the Total RNA Optimization Protocol, the values were obtained using a (1:1) phenol/chloroform solution.

Interestingly, previous studies that isolated miRNAs from saliva neither mentioned the value obtained from the 260/280 ratio nor considered a minimum standard value [11,12,50,52]. According to Lam et al. (2012) [51], plasma, urine, and saliva consistently exhibit a “lower than acceptable” 260/230 ratio, with readings in the range of 2.0–2.2 for the 260/230 ratio being considered pure RNA samples. This study presented values below this range (2.13 ± 0.10), confirming the high purity of the samples. Furthermore, previous studies did not report a minimum value for the purity standard [11,12,53,54,55,56], emphasizing the significance and pioneering nature of the present study.

However, considering the relevant aspects of this study, some limitations should be highlighted, such as the absence of protein characterization by other methods, such as Western blot, as recommended by the EV-TRACK platform (www.evtrack.org accessed on 12 December 2024). The present research evaluated saliva-derived EVs, as saliva is a widely studied biofluid due to its noninvasive collection process and its potential as an excellent source of biomarkers [7]. Therefore, future studies should focus on further characterizing EVs and evaluating the present method using other biofluids.

## 4. Materials and Methods

The Human Ethics Committee of Antônio Pedro University Hospital approved this study (#3.455.574). Informed written consent was obtained from all 44 participants. The study included patients aged 18 to 65 years from Fluminense Federal University, of both genders. Exclusion criteria comprised a history of malignant neoplasm, rheumatoid arthritis, and fibromyalgia. They were randomly selected over a period of 3 months.

### 4.1. Saliva Collection and Preparation

Saliva was collected by rinsing the mouth with 5 mL of physiological saline solution (for one minute). Twenty-seven samples were collected from each subject (participants 1, 2, and 3), totaling 81 samples, with a 24 h interval between collections. Study participants were instructed to refrain from eating, drinking, and smoking and avoid oral hygiene procedures at least 1 h before collection. The collected material was stored at −20 °C until use. First, the saliva was thawed at room temperature and centrifuged at 1500× *g* (Thermo Scientific Centrifuge, Boston, MA, USA) for 10 min at room temperature. Then, the supernatant was transferred to a new 15 mL Falcon tube, discarding the pellet. Next, another centrifugation was performed at 5500× *g* for 20 min at room temperature to remove cell debris [47,52]. The supernatant was then frozen at −80 °C with the tube held upright to ensure consistent freezing and subsequent sublimation during lyophilization according to the manufacturer’s protocol, minimizing sample drying loss.

### 4.2. Isolation of Extracellular Vesicles

For the test group, saliva supernatants from 57 samples were transferred to the drying chamber of an LT1000 lyophilizer (Enterprise II, Terroni Equipment LTDA, São Paulo, Brazil). The lyophilization process achieved a drying rate of approximately 0.8 mm per hour, reflecting the speed at which the sublimation front advanced, increasing the dried layer thickness accordingly. This rate resulted from optimized settings, with temperatures maintained between −35 °C and −41 °C and pressure ranging from 0.03 to 0.2 mbar, tailored for saliva samples, completing the process in about 14 h (overnight). Post-lyophilization, samples were stored at −80 °C until processing, then resuspended in 200 µL of PBS for EV size measurements and electron microscopy. Multiple pre-lyophilization freeze–thaw cycles did not compromise EV or miRNA integrity, aligning with their established stability [10,36].

In the control group (ultracentrifugation), the samples were thawed at room temperature and ultracentrifuged at 160,000× *g* for 2 h at 4 °C using a rotor 70 Ti (Beckman Coulter Ultracentrifuge, Brea, CA, USA). Immediately afterward, the samples were transferred to a 1.5 mL tube. Next, 1 mL of TRIzol^TM^ Reagent (Invitrogen, Carlsbad, CA, USA) was added, and the samples were stored at −80 °C until processing or the pellet was resuspended in 100 μL of PBS for EV size measurements, dot blotting analysis, and electron microscopy. Twenty-four samples were collected from three subjects (1, 2, and 3).

#### Clarification of Lyophilization Purpose

Lyophilization was used to preserve saliva supernatant samples, which included EVs and their molecular cargo, instead of specifically isolating EVs. After freeze-drying, the dry samples were processed for total RNA extraction (Section 4.6), capturing both EV-associated and potentially free RNA, unlike ultracentrifugation, which isolates EV fractions.

### 4.3. Extracellular Vesicle Size Measurements by Quasi-Elastic Light Scattering (QELS)

The effective diameter and the size distribution of EVs suspended in saline were measured in a NanoBrook Omni particle size and zeta potential analyzer (Brookhaven Instruments, Holtsville, NY, USA) at 25 °C, using 5 μL in 1 mL of saline. Multimodal distributions of particle size diameter were generated by a non-negativity-constrained least squares algorithm (NNLS) based on each particle’s light scattering intensity.

### 4.4. Electron Microscopy—Uranyl Acetate Stained Preparations

EVs were deposited onto Formvar-coated grids and allowed to adhere for a minimum of two hours at room temperature. After that, the grids were fixed by inversion on a drop of 4% formaldehyde in PBS for 30 min and stained in a 3% methylcellulose and 4% uranyl acetate solution (9:1) for 10 min on ice. Alternatively, fixed microvesicles were blocked in 150 mM glycine in PBS for 10 min, followed by incubation in PBS containing 2% BSA and 0.01% saponin for 30 min. Afterward, grids were incubated with mouse antibodies anti-CD9 and anti-CD63. (1:100; Thermo Fisher Scientific, Waltham, MA, USA) in a blocking buffer at room temperature for three hours. The samples were then washed in blocking buffer and incubated with 10 nm colloidal gold-conjugated goat antimouse IgG (1:100, in blocking buffer) for one hour before being washed in PBS. Subsequently, the samples were fixed again in 1% glutaraldehyde in PBS for 5 min, washed in water, and stained in a 3% methylcellulose and 4% uranyl acetate solution (9:1) for 10 min on ice. The samples were examined using a JEOL 1200 transmission electron microscope (JCB, Rocester, UK) operating at 80 kV, as described by Pereira et al., 2018 [57]. A negative control omitting primary antibodies was not included in this study; however, the specific localization of gold particles to EV membranes suggests minimal non-specific binding of the secondary antibody.

### 4.5. Dot Blotting Analysis

Twenty-five micrograms of EVs from control (ultracentrifugation) or lyophilized samples were applied to the nitrocellulose membrane. The membrane was blocked with TBS supplemented with 5% non-fat milk and 0.01% Tween for 1 h, followed by incubation with primary antibodies, specifically mouse anti-CD9 or anti-CD63 (1:1000, using the same blocking buffer), for 1 h. After washing and incubation with secondary antibodies conjugated to horseradish peroxidase (1:1500, diluted in blocking buffer), the reactive dots were visualized using SuperSignal West Pico Chemiluminescent Substrate (Thermo Fisher Scientific, Waltham, MA, USA), according to the manufacturer’s instructions. Protein concentration was determined by the Bradford assay, using albumin fraction V as the standard.

### 4.6. Sample Preparation Protocols

The information about the behavior of the lyophilized biofluids in the literature is scarce, and no information was obtained about the lyophilization of biofluids for RNA extraction, as the Trizol Reagent manufacturer’s protocol [58] does not provide guidance on using the reagent for total RNA extraction from biofluids. Therefore, the samples were submitted to several treatments before isolating the total RNA rich in miRNAs. Five protocols for sample preparation were implemented: (1) isolation by the Trizol method with the lyophilized (dry) sample (No Treatment + Trizol Group); (2) addition of 200 µL of RNase-free water to the lyophilized sample, followed by isolation by the Trizol method (H_2_O + Trizol Group); (3) filtration of the salivary supernatant (0.2 µm filter) before lyophilization, and isolation by the Trizol method (Filter + Trizol Group); (4) addition of 200 µL of RNase-free water to the lyophilized sample, freezing at −80 °C for seven days and isolation by the Trizol method (H_2_O + (−80 °C) + Trizol Group); (5) lyophilization combined with keeping the samples at room temperature for 15 days (15 days + Trizol Group) (samples were collected from participants 1, 2, and 3).

For the five sample preparation protocols performed and the control group (ultracentrifugation), the isolation of exosomal RNA was performed using the Trizol method, according to the manufacturer’s protocol for mRNA extraction outlined in the Trizol™ Reagent User Guide (2023) [58] and relevant literature [15,47]. The total RNA concentration was analyzed using a calibrated NanoDrop^®^ Spectrophotometer 2000 (Thermo Scientific, Wilmington, DE, USA) (samples were collected from participants 1, 2, and 3).

### 4.7. Total RNA Optimization Protocol

**Figure 5:**
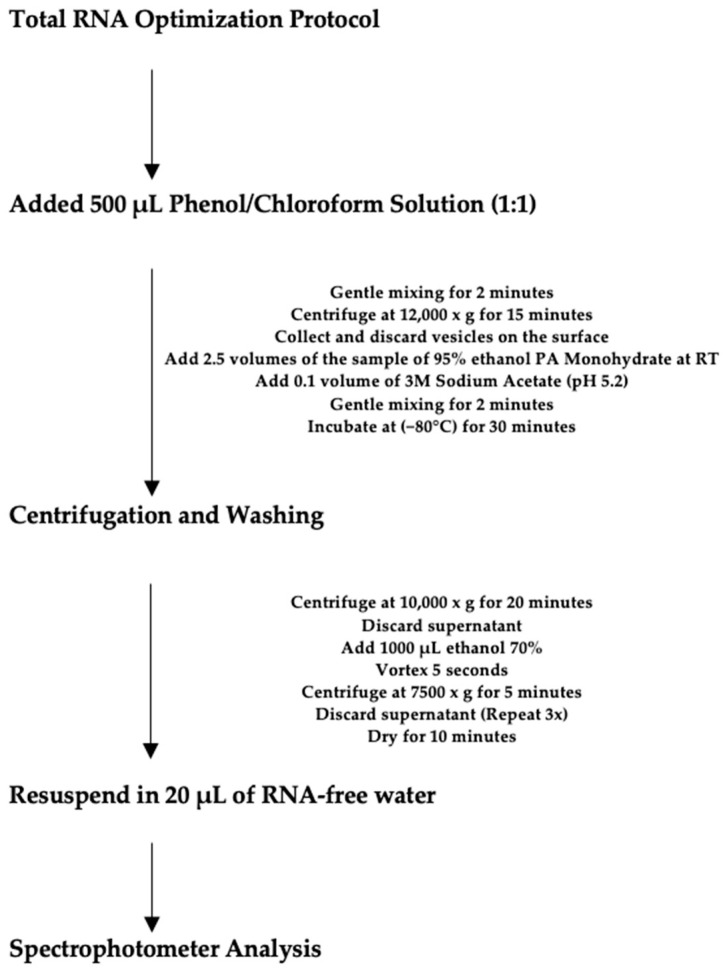
Flowchart demonstrating the step-by-step process of the Total RNA Optimization Protocol.

As the quality of the initial results of the isolated samples, evaluated by the spectrophotometer (ratio 260/280 and 260/230), was below the acceptable references of 1.8 and 2.0, respectively, a purification protocol was developed for the total RNA extracted from saliva supernatant. All isolated total RNA samples underwent a second purification method: adding 500 μL of a 1:1 phenol/chloroform solution to the isolated RNA, gently mixing for 2 min, centrifuging at 12,000× *g* (Sigma, Osterode, Germany) for 15 min at 8 °C, and then collecting and discarding the vesicles formed on the sample’s surface. Subsequently, 2.5 volumes of room temperature 95% ethanol PA Monohydrate were added to the sample, followed by 0.1 volume of 3M Sodium Acetate (pH 5.2). The solution was mixed slowly for 2 min, incubated in a −80 °C freezer for 30 min, and then centrifuged at 10,000× *g* for 20 min at 8 °C. Next, the supernatant was discarded, and 1 mL of 70% ethanol was added, mixed in the vortex, and centrifuged at 7500× *g* for 5 min at 8 °C. The supernatant was discarded again, and this step was repeated three to four times. Finally, the tube was tipped onto absorbent paper for 10 to 15 min to dry the pellet. The pellet was resuspended in 20 μL of RNase-free water, and the total RNA concentration was analyzed using the calibrated NanoDrop^®^ Spectrophotometer 2000 (Thermo Scientific, Wilmington, DE, USA) (Figure 5). The spectrophotometer assessed purity at 230, 260, and 280 nm absorbance. The 260/280 ratio of 1.80 was considered the ideal standard for our samples, based on Lam et al., 2012 [51]. According to the manufacturer’s recommendation, measurements were performed using 1 μL of the extract RNA.

### 4.8. Patient Saliva Supernatant

To make the lyophilization method available for many subject samples, 44 patient samples were collected, lyophilized, isolated, and submitted to the Total RNA Optimization Protocol, forming the Lyophilized Patient Supernatant group.

### 4.9. MicroRNAs Selection

For (q)RT-PCR analyses, microRNA selection was based on a review of previous literature and bioinformatic analysis (URL: http://mirwalk.umm.uni-heidelberg.de accessed on 15 October 2024). One endogenous control (miR-16) and three target miRNAs (miR-21, miR-33a, and miR-146b) were chosen for the microRNA assay.

### 4.10. MicroRNA Analysis Using RT PCR

The TaqMan^®^ MicroRNA Reverse Transcription Kit (PN 4366596, 200 reactions) was used to perform the RT reaction. Next, 0.66 ng/μL of RNA (10 ng), 1X stem-loop RT primer (3 μL for miR-21, miR-33a, and miR-146b), and the endogenous control (miR-16) were added according to the literature [53,59]. A total of 3.33 U/μL reverse transcriptase (1 μL), 0.25 U/μL RNase inhibitor (0.19 μL), 0.25 mM dNTPs (0.15 μL), and 1X reaction buffer (1.50 μL) were combined in a total reaction volume of 15 μL and incubated at 16 °C for 30 min, 42 °C for 30 min, and 85 °C for 5 min in a MyCycler™ thermal cycler (BioArt, Hercules, CA, USA), following the TaqMan^®^ MicroRNA Assays Protocol. Reverse Transcriptase negative samples (RT-, no RT added) were included, along with sample blanks as negative controls (no sample but RT). After the RT step, 1.33 μL of the RT reaction was combined with 1.0 μL of a TaqMan MicroRNA Assay (20X; forward primer, reverse primer, and probe) and 10 μL of TaqMan^®^ Universal PCR Master Mix II, without UNG (PN 4428175), resulting in a final volume of 20 μL. Real-time PCR was performed using an Agilent Technologies Stratagene Mx3005P (Santa Clara, CA, USA), with cycling conditions set at 95 °C for 10 min, followed by 95 °C for 15 s and 60 °C for 60 s, for a total of 40 cycles. Each TaqMan Assay was conducted in duplicate (n = 2 for participants 1 and 2). The C(q) value was considered when assessing the presence of microRNAs in the lyophilized and ultracentrifuged samples.

### 4.11. Statistical Analysis

Data processing and statistical analysis were conducted using GraphPad Prism 9.2.0 (San Diego, CA, USA). The D’Agostino and Pearson test was employed to assess the normality of data distribution. For comparisons between experimental groups, the following statistical tests were utilized: at-Test for two-group comparisons, ANOVA with Tukey’s post hoc test for multiple group comparisons, and the Mann–Whitney test for non-parametric data. Specifically, we compared (1) RNA yields between lyophilization protocols and ultracentrifugation, (2) RNA purity ratios across all preparation methods, and (3) miRNA expression levels between lyophilized and ultracentrifuged samples. Data were expressed as mean and 95% confidence interval (CI), with *p* < 0.05 considered statistically significant.

## 5. Conclusions

Lyophilization is a promising method for isolating extracellular vesicles from saliva supernatant samples. This method can also be useful for isolating mRNA and small RNA, including microRNA, in different biofluids and tissues.

## Figures and Tables

**Table 1:** Comparison of concentration and purity of isolated RNA after ultracentrifugation and lyophilization of saliva samples.

Method	Groups	Concentration ng/µL	260/280	260/230
Ultracentrifugation	Control	110.50 (±11.65)	1.73 (±0.01)	2.18 (±0.10)
Lyophilization	No treatment + Trizol	233.50 (±78.26)	1.82 (±0.04)	2.20 (±0.05)
	H_2_O + Trizol	227.50 (±61.63)	1.82 (±0.05)	2.22 (±0.05)
	Filter + Trizol	227.13 (±89.16)	1.83 (±0.02)	2.24 (±0.02)
	H_2_O + (−80°) + Trizol	237.47 (±43.64)	1.84 (±0.01)	2.13 (±0.10)
	15 days + Trizol	221.01 (±31.50)	1.88 (±0.02)	2.25 (±0.05)
	Patient	196.5 (±103.9)	1.80 (±0.15)	2.18 (±0.19)

**Table 2:** Bioinformatic analysis of miRNA targets.

miRNA	Target Gene	Gene Name
hsa-miR-21 or hsa-miR-21–5p	*TNFRSF11A* or *RANK*	Tumor necrosis factor ligand superfamily member 11A or Receptor activator of nuclear factor κ B
	*STAT5A*	Signal transducer and activator of transcription 5A
has-miR-33a or has-miR-33a-5p	*SATB2*	Special AT-rich sequence-binding protein 2
has-miR-146b or has-miR-146b-5p	*TRAF6*	TNF receptor-associated factor 6
	*STAT3*	Signal transducer and activator of transcription 3
	*RUNX2*	Runt-related transcription factor 2

## Data Availability

Data are contained within the article.
